# Supplementary material for: Graph Peak Caller: Calling ChIP-seq peaks on graph-based reference genomes
Source: PLoS Comput Biol. 2019 Feb 19;15(2):e1006731. doi: 10.1371/journal.pcbi.1006731 (PMC6396939; doi:10.1371/journal.pcbi.1006731)
Supplement: S1 Table — (PDF) [file pcbi.1006731.s001.pdf]

Table S1 Overview of peaks reported by Graph Peak Caller and MACS2 on *Drosophila melanogaster* and human for 5 transcription factors (TFs). In this comparison, the top scoring peaks detected by each peak caller are selected, by for each transcription factor selecting as many peaks as have been detected by the peak caller that is finding the least amount of peaks (*total*). *Shared* are peaks that overlap with a peak from the other peak caller, and *unique* are peaks reported by one peak caller and not the other. The sections *Motif enrichment Graph Peak Caller* and *Motif enrichment MACS2* show the number of shared and unique peaks that have motif match along with the percentages showing the proportion of shared and unique peaks having motif mtach. All peaks have been trimmed to 120 base pairs around the peak summit (position in peak with lowest q-value), to make the comparison clearer.

	Graph Peak Caller					MACS2				
	Total	Shared		Unique		Total	Shared		Unique	
Human										
CTCF	49049	36003/46279	77.80%	959/2770	34.62%	47320	35932/46279	77.64%	391/1041	37.56%
SRF	19123	4711/16640	28.31%	294/2483	11.84%	17963	4706/16640	28.28%	156/1323	11.79%
SUM	68172	40714/62919	64.71%	1253/5253	23.85%	65283	40638/62919	64.59%	547/2364	23.14%
D. m.										
JRA	8717	362/8105	4.47%	23/612	3.76%	8679	359/8105	4.43%	12/574	2.09%
SQZ	10374	1868/9374	19.93%	171/1000	17.10%	10025	1832/9374	19.54%	89/651	13.67%
JIM	5385	1220/4924	24.78%	66/461	14.32%	5607	1215/4924	24.68%	82/683	12.01%
ANTP	7093	94/6409	1.47%	11/684	1.61%	6917	91/6409	1.42%	4/508	0.79%
SUM	31569	3544/28812	12.30%	271/2757	9.83%	31228	3497/28812	12.14%	187/2416	7.74%
