# Supplementary material for: Graph Peak Caller: Calling ChIP-seq peaks on graph-based reference genomes
Source: PLoS Comput Biol. 2019 Feb 19;15(2):e1006731. doi: 10.1371/journal.pcbi.1006731 (PMC6396939; doi:10.1371/journal.pcbi.1006731)
Supplement: S2 Table — (PDF) [file pcbi.1006731.s002.pdf]

Table S2 Average number of base pairs not included in the linear reference genome within peak sets. Here, all peaks have been trimmed to 120 base pairs around the peak summit (position in peak with lowest q-value), to make the comparison clearer.

Average number of base pairs not part of linear reference genome

	Peaks found by Graph Peak Caller that also were found by MACS2	Peaks found uniquely by Graph Peak Caller
A. thaliana		
ERF115	0.863	3.712
SEP3	0.657	2.092
AP1	0.592	2.110
SOC1	0.384	0.917
PI	0.820	2.548
D. melanogaster		
JRA	0.069	0.360
SQZ	0.095	0.408
JIM	0.074	0.426
ANTP	0.075	0.412
Human		
CTCF	0.583	2.385
SRF	0.448	2.274
