# Supplementary material for: Graph Peak Caller: Calling ChIP-seq peaks on graph-based reference genomes
Source: PLoS Comput Biol. 2019 Feb 19;15(2):e1006731. doi: 10.1371/journal.pcbi.1006731 (PMC6396939; doi:10.1371/journal.pcbi.1006731)
Supplement: S3 Table — (PDF) [file pcbi.1006731.s003.pdf]

Table S3. Similar to Table 1 in the manuscript, but instead of MACS2 using reads aligned by BWA, it uses vg alignments projected to the linear reference genome.

	Graph Peak Caller					MACS2				
	Total	Shared		Unique		Total	Shared		Unique	
ERF115	24976	3121/21364	14.61%	284/3612	7.86%	23167	3108/21364	14.55%	214/1803	11.87%
SEP3	14976	989/11982	8.25%	125/2994	4.18%	15517	978/11982	8.16%	148/3535	4.19%
AP1	16797	764/13405	5.70%	102/3392	3.01%	17030	754/13405	5.62%	94/3625	2.59%
SOC1	15502	1676/14297	11.72%	116/1205	9.63%	16407	1681/14297	11.76%	142/2110	6.73%
PI	17518	1790/14084	12.71%	300/3434	8.74%	16084	1793/14084	12.73%	152/2000	7.60%
SUM	89769	8340/75132	11.10%	927/14637	6.33%	88205	8314/75132	11.07%	750/13073	5.74%
