# Supplementary figures and images for: Graph Peak Caller: Calling ChIP-seq peaks on graph-based reference genomes
Source: PLoS Comput Biol. 2019 Feb 19;15(2):e1006731. doi: 10.1371/journal.pcbi.1006731 (PMC6396939; doi:10.1371/journal.pcbi.1006731)

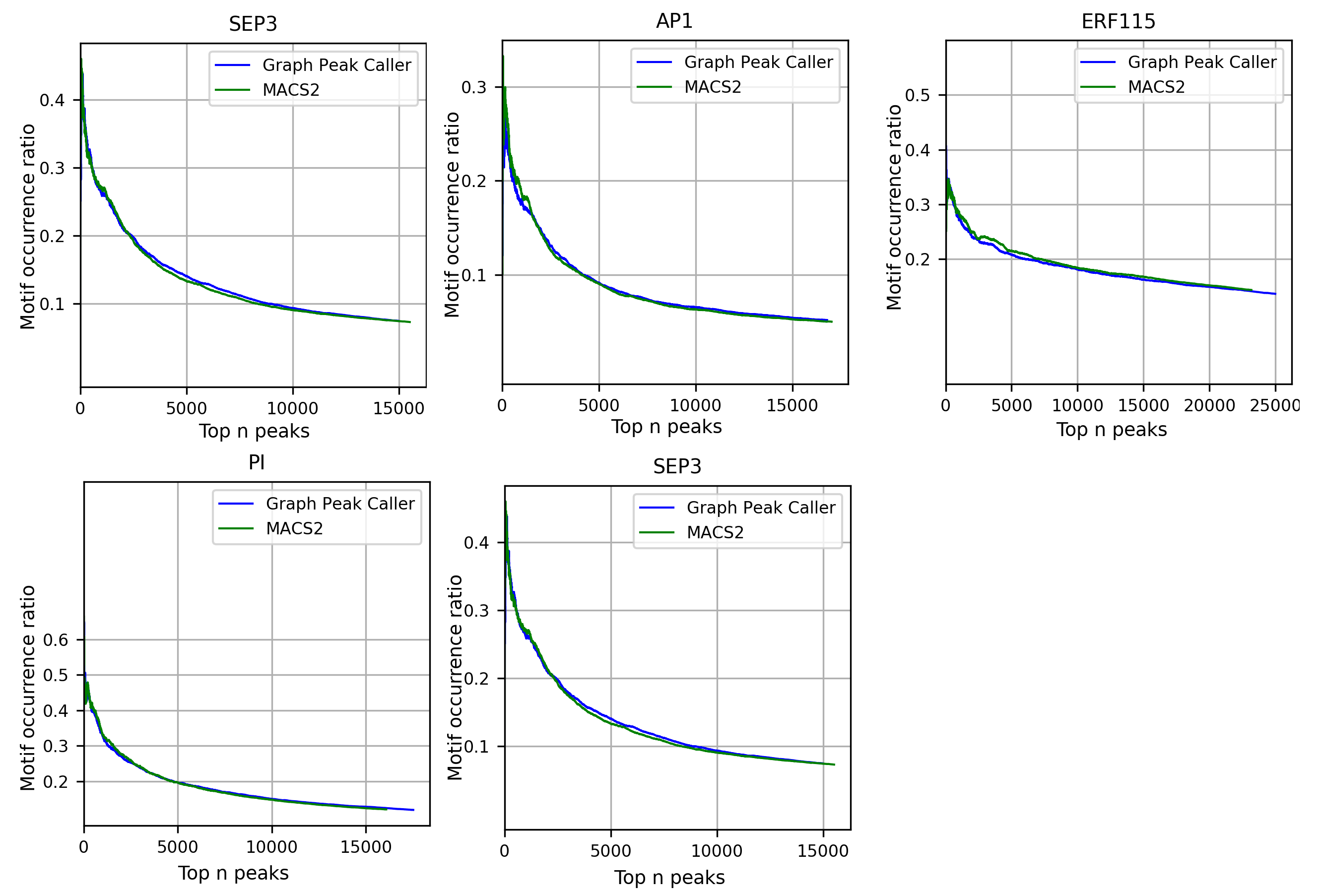

Supplement: S1 Fig — Contrary to Fig 5, these plots include all peaks found by both peak callers. (TIF) [file pcbi.1006731.s004.tif]

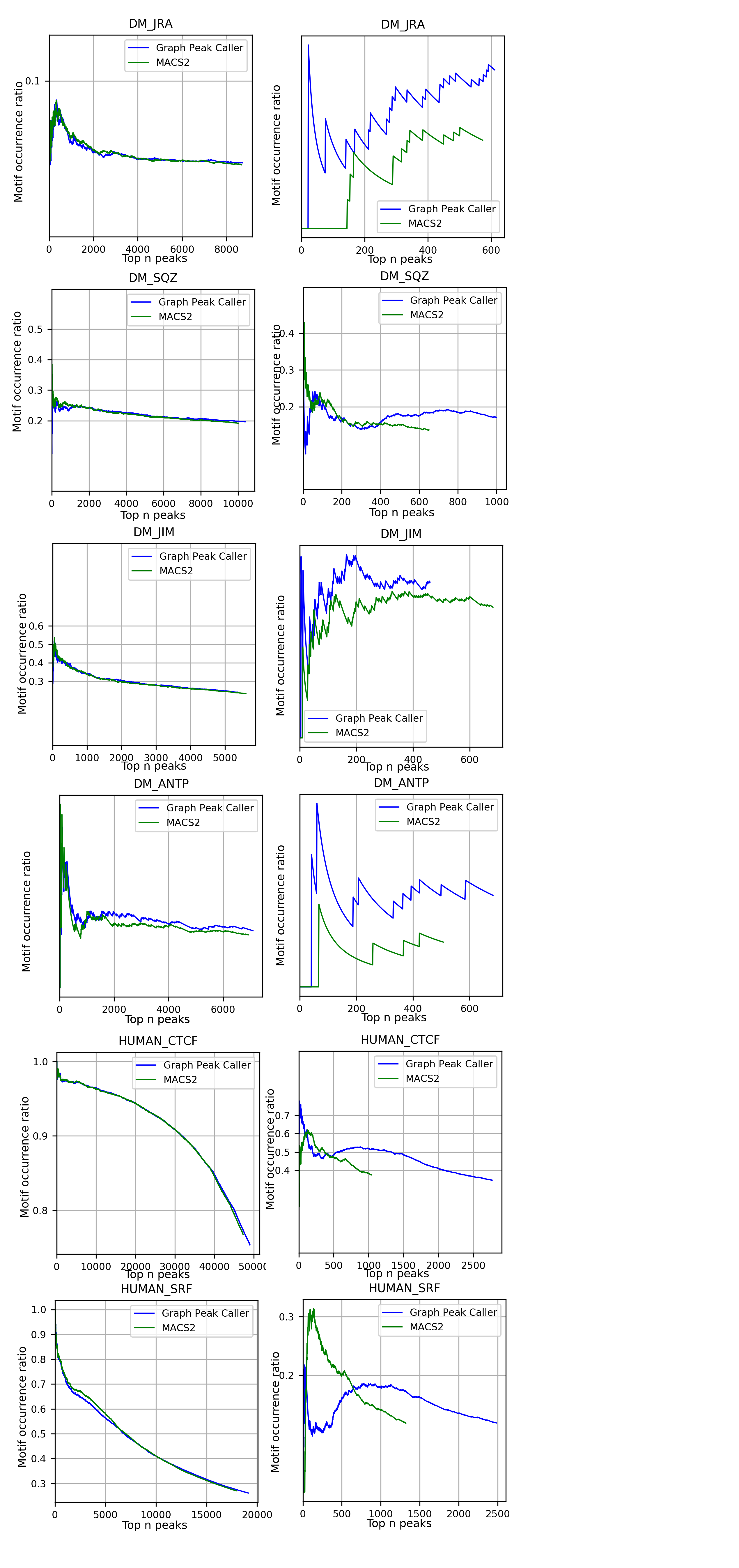

Supplement: S2 Fig — Left plots are proportion of peaks matching motif when all peaks are included. Right plots are proportion of peaks matching motif when only unique peaks found by each peak caller are included. (TIF) [file pcbi.1006731.s005.tif]

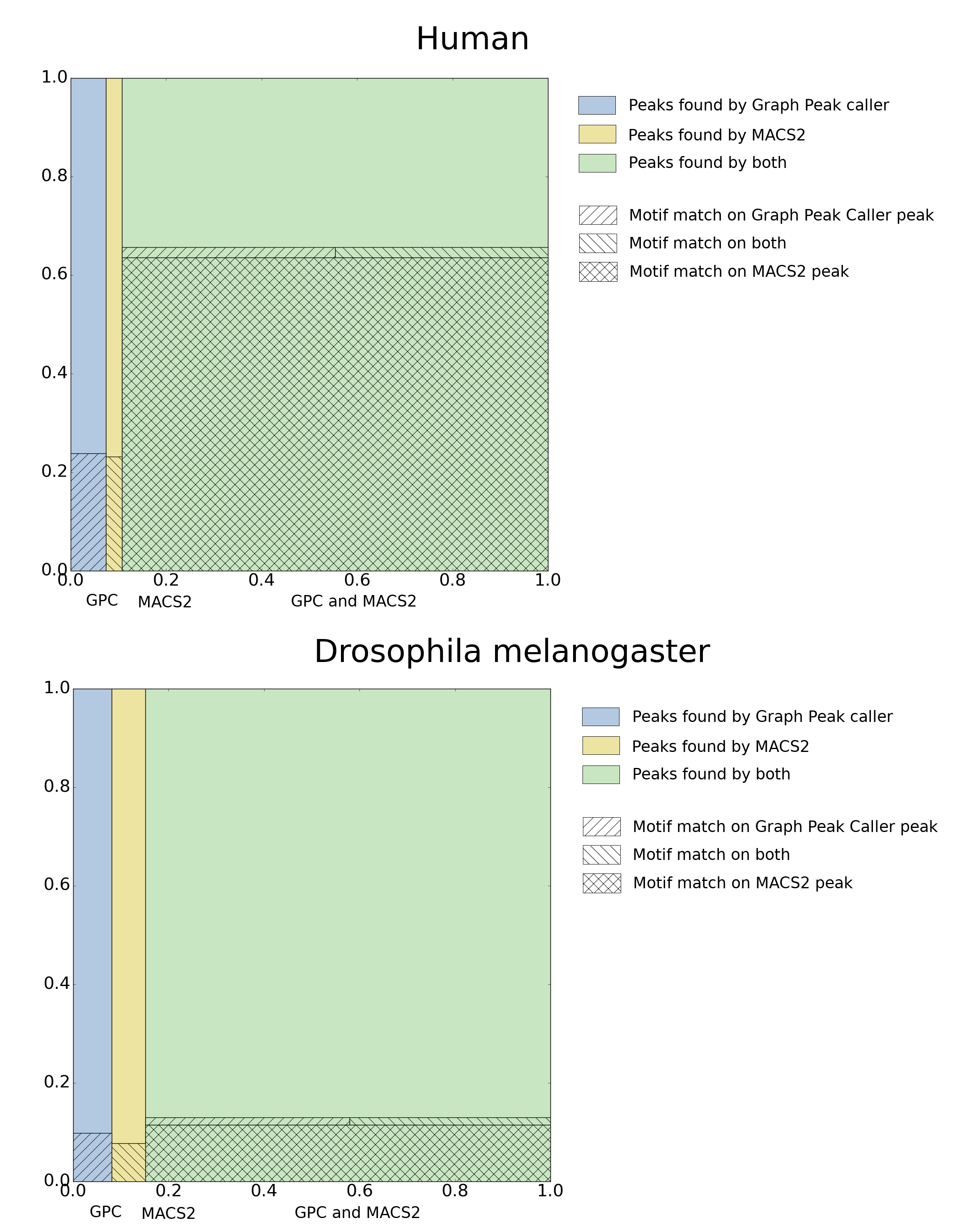

Supplement: S3 Fig — (TIF) [file pcbi.1006731.s006.tif]
