# Supplementary material for: Graph Peak Caller: Calling ChIP-seq peaks on graph-based reference genomes
Source: PLoS Comput Biol. 2019 Feb 19;15(2):e1006731. doi: 10.1371/journal.pcbi.1006731 (PMC6396939; doi:10.1371/journal.pcbi.1006731)
Supplement: S2 Appendix — List of fastq IDs and resulting alignment sequences intersecting with the binding site shown in Fig 3, showing that all of these appear in the same haplotype. (PDF) [file pcbi.1006731.s008.pdf]

S2 Appendix - Reads behind Figure 3

The following fastq reads overlap with the region shown in Figure 3 (reads ID from fastq with SRA ID SRR931836).

SRR931836.531994, SRR931836.952533, SRR931836.1053622, SRR931836.1069887, SRR931836.1420071, SRR931836.1569407, SRR931836.2175300, SRR931836.2611801, SRR931836.2851952, SRR931836.3202289, SRR931836.3224927, SRR931836.4770490, SRR931836.5407974, SRR931836.6352336, SRR931836.7388866, SRR931836.7505309, SRR931836.7713938, SRR931836.7918128, SRR931836.8527838, SRR931836.8677342, SRR931836.9036755, SRR931836.9101957, SRR931836.9526182, SRR931836.9557079, SRR931836.9597616, SRR931836.10067214, SRR931836.11485357, SRR931836.11791390, SRR931836.11811808, SRR931836.12373570, SRR931836.14154848, SRR931836.14313085, SRR931836.14502580, SRR931836.16566511, SRR931836.16928475, SRR931836.17213611, SRR931836.17405600

These reads result in alignments with the following sequences when aligned to the A*.* *thaliana* reference graph (sequences are in same order as the read IDs above):

catctaccttgtttttctttgacggcgacatacaaaaaataaaacaaaac
ccttcttcttcatctaccttgtttttctttgacggcgacatacaaaaaat
catctaccttgtttttctttgacggcgacatacaaaaaataaaacaaaac
cttcttcttcatctaccttgtttttctttgacggcgacatacaaaaaat
aatccattttctcgttttcgatgtccttcttcttcatctaccttgttttt
gacatacaaaaaataaaacaaaacagaaccctagatgggtgtgatgggct
tctcgttttcgatgtccttcttcttcatctaccttgtttttctttgacgg
ttttctcgttttcgatgtccttcttcttcatctaccttgtttttctttg
gacggcgacatacaaaaaataaaacaaaacagaaccctagatgggtgtg
tcttcatctaccttgtttttctttgacggcgacatacaaaaaataaaa
ccattttctcgttttcgatgtccttcttcttcatctaccttgtttttct
gacggcgacatacaaaaaataaaacaaaacagaaccctagatgggtgtga
gttttcgatgtccttcttcttcatctaccttgtttttctttgacggcgac
gacggcgacatacaaaaaataaaacaaaacagaaccctagatgggtgtg
ttctcgttttcgatgtccttcttcttcatctaccttgtttttctttgacg
tctcgttttcgatgtccttcttcttcatctaccttgtttttctttgacgg
ctaccttgtttttctttgacggcgacatacaaaaaataaaacaaaac
gttttcgatgtccttcttcttcatctaccttgtttttctttgacggcgac
ctaccttgtttttctttgacggcgacatacaaaaaataaaacaaaac
cgatgtccttcttcttcatctaccttgtttttctttgacggcgacataca
cgttttcgatgtccttcttcttcatctaccttgtttttctttgacggcga
ccttcttcttcatctaccttgtttttctttgacggcgacatacaaaaaat
ccttgtttttctttgacggcgacatacaaaaaataaaacaaaacagaacc
tctcgttttcgatgtccttcttcttcatctaccttgtttttctttgacgg
ttttctttgacggcgacatacaaaaaataaaacaaaacagaaccct
gtttttctttgacggcgacatacaaaaaataaaacaaaacagaaccta
accttgtttttctttgacggcgacatacaaaaaataaaacaaaacagaac
catctaccttgtttttctttgacggcgacatacaaaaaataaaacaaaac
tttttctttgacggcgacatacaaaaaataaaacaaaacagaaccct
atgtccttcttcttcatctaccttgtttttctttgacggcgacatacaa
ttgacggcgacatacaaaaaataaaacaaaacagaaccctagatgggtg
gatgtccttcttcttcatctaccttgtttttctttgacggcgacatacaa
cacaaatccattttctcgttttcgatgtccttcttcttcatctaccttgt
atccattttctcgttttcgatgtccttcttcttcatctaccttgtttttc
tttgacggcgacatacaaaaaataaaacaaaacagaaccctagatgggtg
ttttctttgacggcgacatacaaaaaataaaacaaaacagaaccctaga
cgttttcgatgtccttcttcttcatctaccttgtttttctttgacggcga

This is the sequence on haplotype 628 from the 1001 genomes vcf (http://1001genomes.org/data/GMI-MPI/releases/v3.1/1001genomes_snp-short-indel_only_ACGTN.vcf.gz) used to build the graph:

CACAAATCCATTTTCTCGTTTTCGATGTCCTTCTTCTTCATCTACCTTGTTTTTCTTTGACGGCGACATACAAAAAATAAAACAAAACAGAACCCTAGATGGGTGTGATGGGCT

All above sequences are found within this sequence, either direct or in reverse complement.
