# Supplementary material for: Graph Peak Caller: Calling ChIP-seq peaks on graph-based reference genomes
Source: PLoS Comput Biol. 2019 Feb 19;15(2):e1006731. doi: 10.1371/journal.pcbi.1006731 (PMC6396939; doi:10.1371/journal.pcbi.1006731)
Supplement: S3 Appendix — (PDF) [file pcbi.1006731.s009.pdf]

S3 Appendix

Alignment motif enrichment:

Sequences for alignments were extracted from the reference and Fimo was used to find motif-matches, with the same parameters as when evaluating the peak callers:

	ERF115	SOC1	SEP3	AP1	PI	Aggr
Alignments MACS2	10800502	2871375	7888164	8430747	9807817	39798605
Alignments GPC	11472730	3587887	8592532	9021976	9994855	42669980
Motif matches MACS2	331839	45023	132673	161919	241856	913310
Motif matches GPC	348812	47861	139799	172421	248433	957326
Motif rate MACS2	0.0307	0.0157	0.0168	0.0192	0.0247	0.0214
Motif rate GPC	0.0304	0.0133	0.0163	0.0191	0.0249	0.0208

Haplotype analysis:

Variants were found by mapping node ids to VCF-file variants. An interval is considered consistent with a haplotype in the vcf if all the variants included in the interval is present in the haplotype.

Haplotype consistency of motif-intervals:

Here we extracted the graph interval corresponding to all the motif-matches on the graph peak caller predicted peaks, and checked whether the interval was consistent with a haplotype in the vcf. The table shows [n_haplotype_consistent_motifs/n_total_motifs haplotype_consistent_ratio]

	ERF115	PI	SOC1	AP1	SEP3	Aggr
Unique	284/284<br>1.0	296/300<br>0.9867	113/116<br>0.9741	98/102<br>0.9608	123/125<br>0.9840	**914/927<br>0.9860**
Shared	3119/3121<br>0.9994	1777/1790<br>0.9927	1673/1676<br>0.9982	760/764<br>0.9948	986/989<br>0.9970	8315/8340<br>0.9970
Aggr	3403/3405<br>0.9994	2073/2090<br>0.9919	1786/1792<br>0.9967	858/866<br>0.9908	1109/1114<br>0.9955	**9229/9267<br>0.9959**

Removing all motif-matches that doesn't correspond to a single haplotype gives the ratio of matches on unique peaks 914/14637=0.624, which is still significantly better than for Macs, p=3.80%.

Motifs on diplotype-consistent peaks:

Here we filtered out all peaks from graph peak caller that didn't have diplotype consistent alignments. To be diplotype consistent we required that all alignments that overlap with the peak were compatible with either of two haplotypes from the vcf:

	ERF115	PI	SOC1	AP1	SEP3	Aggr
Number of peaks	1987	2320	396	1955	1475	8133
Number of peaks with motif match	179	204	32	51	50	516
Ratio of peaks with motif match	0.0901	0.0879	0.0808	0.0261	0.0339	**0.0634**

Only considering diploid-consistent peaks gives a motif-match ratio of 0.0634, which is still significantly higher then for MACS (p=3.47%)

Diplotype effect compared with MACS

Here we checked the ratio of motif matches on diplotype-consistent peaks and not diplotype-consistent peaks for both MACS and GPC. We see that the difference in motif ratio between diploid- and not-diploid-consistent peaks are very similar for MACS and GPC peaks

	ERF115	PI	SOC1	AP1	SEP3
MACS-DIP	0.1310	0.0708	0.0597	0.0270	0.0396
MACS-NOT-DIP	0.0793	0.1034	0.0930	0.0215	0.0504
**MACS-DIFF**	**0.0518**	**-0.0327**	**-0.0333**	**0.0055**	**-0.0108**
GPC-DIP	0.0900	0.0879	0.0808	0.0261	0.0339
GPC-NOT-DIP	0.0793	0.1034	0.0930	0.0215	0.0504
**GPC-DIFF**	**0.0255**	**0.0018**	**-0.0230**	**-0.0094**	**-0.0155**
